# Domain adaptation of statistical machine translation with domain-focused web crawling

**DOI:** 10.1007/s10579-014-9282-3

**Published:** 2014-12-03

**Authors:** Pavel Pecina, Antonio Toral, Vassilis Papavassiliou, Prokopis Prokopidis, Aleš Tamchyna, Andy Way, Josef van Genabith

**Affiliations:** 1 Charles University in Prague, Prague, Czech Republic; 2 Dublin City University, Dublin, Ireland; 3 Institute for Language and Speech Processing/Athena RIC, Athens, Greece; 4 Universität des Saarlandes, 66123 Saarbrücken, Germany; 5 DFKI, German Research Center for Artificial Intelligence, 66123 Saarbrücken, Germany

**Keywords:** Statistical machine translation, Domain adaptation, Web crawling, Optimisation

## Abstract

In this paper, we tackle the problem of domain adaptation of statistical machine translation (SMT) by exploiting domain-specific data acquired by domain-focused crawling of text from the World Wide Web. We design and empirically evaluate a procedure for automatic acquisition of monolingual and parallel text and their exploitation for system training, tuning, and testing in a phrase-based SMT framework. We present a strategy for using such resources depending on their availability and quantity supported by results of a large-scale evaluation carried out for the domains of environment and labour legislation, two language pairs (English–French and English–Greek) and in both directions: into and from English. In general, machine translation systems trained and tuned on a general domain perform poorly on specific domains and we show that such systems can be adapted successfully by retuning model parameters using small amounts of parallel in-domain data, and may be further improved by using additional monolingual and parallel training data for adaptation of language and translation models. The average observed improvement in BLEU achieved is substantial at 15.30 points absolute.

## Introduction

Recent advances in statistical machine translation (SMT) have improved machine translation (MT) quality to such an extent that it can be successfully used in industrial processes (e.g., Flournoy and Duran 2009). However, this mostly happens only in specific domains where ample training data is available (e.g., Wu et al. 2008). Using in-domain data for training has a substantial effect on the final translation quality: the performance of an SMT system usually drops when it is applied to data of a different nature than that on what it was trained (e.g., Banerjee et al. 2010).

SMT is an instance of a machine learning application which in general works best if the data for training and testing are drawn from the same distribution (i.e., domain, genre, and style). In practice, however, it is often difficult to obtain sufficient amounts of in-domain data (in particular, parallel data required for translation and reordering models) to train a system with good performance for a specific domain. The main problem is usually vocabulary coverage: domain-specific texts typically contain a substantial amount of special vocabulary, which is not likely to be found in texts from other domains (Banerjee et al. 2010). Additional problems can be caused by divergence in style or genre, where the difference is not only in lexis but also in other linguistic aspects such as grammar.

In order to achieve optimal performance, an SMT system should be trained on data from the same domain, genre, and style as it is intended to be applied to. For many domains, though, in-domain data of a sufficient size to train an SMT system with good performance is difficult to find. Recent experiments have shown that even small amounts of such data can be used to adapt an existing (general-domain) system to the particular domain of interest (Koehn et al. 2007). Sometimes, appropriate sources of such data come in the form of existing in-house databases and translation memories (He et al. 2010). An alternative option pursued in this paper is to exploit the constantly growing amount of publicly available text on the web, although acquiring data of a sufficient quality and quantity from this resource is a complicated process involving several critical steps (crawling, language identification, cleaning, etc.).

In this research, we first present a strategy and relevant workflows for automatic web-crawling and cleaning of domain-specific data with limited manual intervention. These workflows are based on open-source tools and have also been deployed as web services in the context of the Panacea^1^ research project (Poch et al. 2012). One advantage of making the tools available as services is that chaining them together enables the building of dynamic and flexible workflows, which can always be improved by integrating new services and/or old legacy systems that may run on different technological platforms. Moreover, the user does not have to deal with technical issues regarding the tools, such as their installation, configuration, or maintenance.

These workflows are then employed to acquire monolingual and parallel data for two domains: environment (*env*) and labour legislation (*lab*), and two language pairs: English–French (EN–FR) and English–Greek (EN–EL). The crawled data is further exploited for domain adaptation of a general-domain SMT system in several ways: by domain-specific parameter tuning of the main log-linear model and by adaptation of its components. The evaluation experiments carried out in a total of eight evaluation scenarios (two domains, two language pairs, and both translation directions: to and from English) confirm substantial and consistent improvements in translation quality for all approaches compared to the baseline.

We explain the improvements brought about by analysing the experimental results in detail. In a nutshell, tuning for matching-domain training and test data results in weight vectors that trust (often long) translation table entries. Tuning with and for specific domains (while using generic training data) allows the MT system to stitch together translations from smaller fragments which, in this case, leads to improved translation quality. Such tuning requires only small development sets which can be harvested automatically from the web with minimal human intervention; no manual cleaning of the development data is necessary.

In addition, additional improvements are realised by using monolingual and/or parallel in-domain training data. Adaptation of language models focuses on improving translation fluency and lexical selection for the particular domain. Adaptation of the translation model then aims at reduction of the out-of-vocabulary (OOV) rate and adding domain-relevant translation variants. All the data sets are available via the European Language Resources Association (ELRA).

This paper is an extended and updated version of our previous work published as Pecina et al. (2011, 2012a, b). Compared to these conference papers, we provide more details of the experiments, full results and a more thorough analysis and description of our findings. Some experiments are new, and not contained in the standalone papers. These include a comparison of various methods for adaptation of language models and translation models (including the state-of-the-art linear interpolation), as well as the comparison of OOV rate (i.e., the ratio of source words unknown to the translation model), language model perplexity measures, and average phrase length in the test set translations (cf. Table 15). Compared to the previous papers, the translation quality evaluation in this work is conducted on tokenized and lowercased translations to avoid any bias caused by recasing and detokenization. We also provide much longer descriptions of both related work as well as our data acquisition procedure. Finally, we formulate this paper as one concise yet coherent account of the full range of experiments carried out.

The remainder of the paper is organised as follows. After the overview of related work and description of the state-of-the-art in Sect. 2, we present our web-crawling procedure for monolingual and parallel data in Sect. 3, and the baseline system including its evaluation in Sect. 4. Section 5 is devoted to system adaptation by parameter tuning and Sect. 6 to adaptation of language and translation models. Section 7, which concludes the work, is followed by an Appendix containing formal definitions of the two domains relevant to our work and complete results of the main experiments.

## State-of-the-art and related work

In this section, we review the current state-of-the-art in the area of web crawling for monolingual as well as parallel data and briefly describe the main concepts of phrase-based SMT (PB-SMT) and its adaptation to specific domains.

### Web crawling for textual data

Web crawling is the automatic process of travelling through the World Wide Web by extracting links of already fetched web pages and adding them to the list of pages to be visited. The selection of the next link to be followed is a key challenge for the evolution of the crawl and is tied to the goal of the process. For example, a crawler that aims to index the web as a whole may not prioritise the links at all, while a focused/topical crawler that aspires to build domain-specific web collections (Qin and Chen 2005) may use a relevance score to decide which pages to visit first or not at all.

Several algorithms have been exploited for selecting the most promising links. The Best-First algorithm (Cho et al. 1998) sorts the links with respect to their relevance scores and selects a predefined amount of them as the seeds for the next crawling cycle. The PageRank (Brin and Page 1998) algorithm exploits the ‘popularity’ of a web page, i.e., the probability that a random crawler will visit that page at any given time, instead of its relevance. Menczer and Belew (2000) propose an adaptive population of agents and search for pages relevant to a domain using evolving query vectors and neural nets to decide which links to follow.

In other approaches (Dziwiński and Rutkowska 2008; Gao et al. 2010), the selection of the next links is also influenced by the distance between relevant pages (i.e., the number of links the crawler must follow in order to visit a particular page starting from another relevant page). A general framework, which defines crawling tasks of variable difficulty and fairly evaluates focused crawling algorithms under a number of performance metrics (precision and recall, relevance, algorithmic efficiency, etc.) was proposed by Srinivasan et al. (2005).

Another challenging task in producing good-quality language resources from the web is the removal of parts of the web page such as navigation links, advertisements, disclaimers, etc. (often called boilerplate), since they are of only limited or no value for the purposes of studying language use and change (Kilgarriff and Grefenstette 2003) or for training an MT system. A review of cleaning methods is presented by Spousta et al. (2008), among others.

Apart from the crawling algorithm, classification of web content as relevant or otherwise affects the acquisition of domain-specific resources, on the assumption that relevant pages are more likely to contain links to more pages in the same domain. Qi and Davison (2009) review features and algorithms used in web page classification. Most of the reviewed algorithms apply supervised machine-learning methods (support vector machines, decision trees, neural networks, etc.) on feature vectors consisting of on-page features, such as textual content and HTML tags (Yu et al. 2004). Many algorithms exploit additional information contained in web pages, including anchor text of hyperlinks. Some methods adopt the assumption that neighbouring pages are likely to be in the same domain (Menczer 2005).

The WebBootCat toolkit (Baroni et al. 2006) harvests domain-specific data from the web by querying search engines with tuples of in-domain terms. Combine^2^ is an open-source focused crawler based on a combination of a general web crawler and a topic classifier. Efficient focused web crawlers can be built by adapting existing open-source frameworks such as Heritrix,^3^ Nutch,^4^ and Bixo.^5^

### Web crawling for parallel texts

Compared to crawling for monolingual data, acquisition of parallel texts from the web is even more challenging. Even though there are many multilingual websites with pairs of pages that are translations of each other, detection of such sites and identification of the pairs is far from straightforward.

Considering the web as a parallel corpus, Resnik and Smith (2003) present the STRAND system, in which they use a search engine to search for multilingual websites and examine the similarity of the HTML structures of the fetched web pages in order to identify pairs of potentially parallel pages. Besides structural similarity, systems such as PTMiner (Nie et al. 1999) and WeBiText (Désilets et al. 2008) filtered fetched web pages by keeping only those containing language markers in their URLs. Chen et al. (2004) proposed the Parallel Text Identification System, which incorporated a content analysis module using a predefined bilingual wordlist. Similarly, Zhang et al. (2006) adopted a naive aligner in order to estimate the content similarity of candidate parallel web pages. Esplà-Gomis and Forcada (2010) developed Bitextor, a system combining language identification with shallow features (file size, text length, tag structure, and list of numbers in a web page) to mine parallel pages from multilingual sites that have been already been stored locally with the HTTrack^6^ website copier. Barbosa et al. (2012) crawl the web and examine the HTML DOM tree of visited web pages with the purpose of detecting multilingual websites based on the collation of links that are very likely to point to in-site pages in different languages. Once a multilingual site is detected, they use an intra-site crawler and alignment procedures to harvest parallel text for multiple pairs of languages.

### Phrase-based statistical machine translation

In PB-SMT (e.g., Moses (Koehn et al. 2007)), an input sentence is segmented into sequences of consecutive words, called phrases. Each phrase is then translated into a target-language phrase, which may be reordered with other translated phrases to produce the output.

Formally, the model is based on the noisy channel model. The translation ${\mathbf e}$ of an input sentence ${\mathbf f}$ is searched for by maximising the translation probability $p({\mathbf e} |{\mathbf f} )$ formulated as a log-linear combination of a set of feature functions $h_i$ and their weights $\lambda _i$:

$$\begin{aligned} p({\mathbf e} |{\mathbf f} ) = \prod _{i=1}^n h_i({\mathbf e} ,{\mathbf f} )^{\lambda _i}. \end{aligned}$$

Typically, the components include features of the following models (the symbols in brackets refer to the actual features used in our experiments described in Sects. 4–6): *reordering* (*distortion*) *model* ($h_1$–$h_7$), which allows the reordering of phrases in the input sentences (e.g., distance-based and lexicalised reordering), *language model* ($h_8$), which ensures that the translations are fluent, *phrase translation model* ($h_8$–$h_{12}$), which ensures that the source and target phrases are good translations of each other (e.g., direct and inverse phrase translation probability, direct and indirect lexical weighting, and phrase penalty), *phrase penalty* ($h_{13}$), which controls the number of phrases the translation consists of, and *word penalty* ($h_{14}$), which prevents the translations from being too long or too short.

The weights of the log-linear combination influence overall translation quality; however, the optimal setting depends on the translation direction and data. A common solution to optimise weights is to use Minimum Error Rate Training (MERT: Och 2003), which automatically searches for the values that minimise a given error measure (or maximise a given translation quality measure) on a development set of parallel sentences. Theoretically, any automatic measure can be used for this purpose; however, the most commonly used is BLEU (Papineni et al. 2002). The search algorithm is a type of coordinate ascent: considering the $n$-best translation hypotheses for each input sentence, it updates the feature weight which is most likely to improve the objective and iterates until convergence. The error surface is highly non-convex. Since the algorithm cannot explore the whole parameter space, it may converge to a local maximum. In practice, however, it often produces good results (Bertoldi et al. 2009).

### Domain adaptation in statistical machine translation

Domain adaptation is a very active research topic within the area of SMT. Three main topics can be identified depending on the availability of domain-specific data: (1) if any in-domain data is available, it can be directly used to improve the MT system, e.g., by combining the (limited) in-domain with (more extensive) out-of-domain resources for training; (2) if in-domain data exists but is not readily available, one may attempt to acquire domain-specific data (e.g., from the web, which is the case of our work); (3) finally, if sources of in-domain data cannot be identified, one may attempt to select pseudo in-domain data (Axelrod et al. 2011) from general-domain sources. Below, we review a selection of relevant work that falls into these topics.

The first attempt to perform domain adaptation was carried out by Langlais (2002), who integrated in-domain lexicons in the translation model. Wu and Wang (2004) used in-domain data to improve word alignment in the training phase. Much work on domain adaptation in the interim has looked at mixture modelling, whereby separate models are built for each data set (e.g., in-domain and out-of-domain) which are then interpolated. There have been attempts to combine both language models (Koehn and Schroeder 2007) and translation models (Nakov 2008; Sanchis-Trilles and Casacuberta 2010; Bisazza et al. 2011). The features of the different models can be combined by linear or log-linear interpolation (Foster and Kuhn 2007; Banerjee et al. 2011). Ways to optimize interpolation weights include the minimization of the model perplexity on a development set (Sennrich 2012) and the maximization of an evaluation metric (Haddow 2013). Mixture model techniques have been applied to a number of scenarios, including the combination of different kinds of data (e.g., questions and declarative sentences, Finch and Sumita 2008) and the combination of different types of translation models (e.g., surface form and factored, Koehn and Haddow 2012).

A second strand towards domain adaptation regards the acquisition of in-domain data. Munteanu and Marcu (2005) extract in-domain sentence pairs from comparable corpora. Daumé III and Jagarlamudi (2011) attempt to reduce OOV terms when targeting a specific domain by mining their translations from comparable corpora. Bertoldi and Federico (2009) rely on large amounts of in-domain monolingual data to create synthetic parallel corpora for training. Pecina et al. (2011) exploit automatically web-crawled in-domain resources for parameter optimisation and to improve language models. Pecina et al. (2012a) extend this work by using the web-crawled resources to also improve translation models.

The selection of pseudo in-domain data is another approach to domain-adaptation based on the assumption that a sufficiently broad general-domain corpus will include sentences that resemble the target domain. Eck et al. (2004) present a technique for adapting the language model by selecting similar sentences from available training data. Hildebrand et al. (2005) extended this approach to the translation model. Foster et al. (2010) weight phrase pairs from out-of-domain corpora according to their relevance to the target domain. Moore and Lewis (2010) used difference of cross-entropy given an in-domain model and general-domain model to filter monolingual data for language modelling. Axelrod et al. (2011) used a similar approach to filter parallel training data. Recent works extend the cross-entropy approach by combining this score with scores based on quality estimation (Banerjee et al. 2013) and translation models (Mansour et al. 2011) and by using linguistic units instead of surface forms to perform the selection (Toral 2013).

In a recent workshop held to better understand and address issues that arise in domain adaptation for MT (Carpuat et al. 2012), the use of phrase-sense disambiguation (Carpuat and Wu 2007) to model content in SMT was investigated, with the conclusion that it can successfully model lexical choice across domains. In addition, a method for translation mining based on document-pair marginal matching was developed, with the aim of acquiring useful translations for OOVs from comparable and parallel data.

## Domain-focused web crawling for monolingual and parallel data

Domain-focused web crawling aims to visit (and store) web pages relevant to a specific domain only. A critical issue is the construction of the domain definition (see Appendix), since each web page visited by the crawler should be classified as relevant or non-relevant to the domain with respect to this definition. As we did not possess training data for the domains and languages targeted in our experiments, we followed the approach of Ardö and Golub (2007) and represented each domain as a list of weighted terms. Formally, the domain definition consists of triplets 〈*relevance weight, term, domain or subdomain(s)*〉. If the terms are publicly available online, as is often the case, this approach does not require any domain expertise.

For our experiments, we selected English, French, and Greek terms (both single- and multi-word entries) from the “Environment” (536, 277, and 513 terms respectively) and “Employment and Working Conditions” (134, 96, and 157 terms respectively) domains of the EuroVoc^7^ thesaurus v4.3. The EuroVoc structure also allowed us to automatically assign each term to one or more of the following subdomains: natural environment, deterioration of the environment, environmental policy, energy policy and cultivation of agricultural land for *env*; labour law and labour relations, organisation of work and working conditions, personnel management and staff remuneration, employment and labour market for *lab*. Information about subdomains can prove useful in acquiring more focused collections.

**Table 1:** An extract of an example English definition manually constructed for the environment domain

Weight	Term	Subdomain(s)
80	Desertification	Deterioration of the environment; natural environment
80	Available energy resources	Energy policy; natural environment
100	Biodiversity	Natural environment
50	Clean industry	Environmental policy
70	Deforestation	Cultivation of agricultural land; deterioration of the environment
−100	Music	

Each entry was manually assigned a weight indicating the term’s domain relevance, with higher values denoting more relevant terms. Even though a domain expert is required to differentiate relevant terms and assign various weights to them, initial experiments showed that a domain-specific corpus can be constructed (see Sect. 3.1) by using a unique positive weight at the scale of 100. In case of ambiguous terms (e.g., “heavy metal” as a music genre and as an element dangerous for the environment), a user could either exclude this term from the domain definition or assign a negative weight to a term closely related to the ambiguous term’s unwanted reading (i.e., include the term “music” and assign it a negative weight) in order to penalize occurrences of this term. For illustration, a sample from the definition for the *env* domain is given in Table 1.

### Acquisition of monolingual texts

In order to acquire in-domain corpora from the web, we implemented an open-source focused crawler (Papavassiliou et al. 2013). The crawler adopts a distributed computing architecture based on Bixo, an open-source web-mining toolkit running on top of Hadoop^8^ and making use of ideas from the Nutch and Heritrix web crawlers. In addition, the crawler integrates procedures for normalisation, language identification, boilerplate removal, text classification and URL ranking. Users can configure several settings related to focused crawling (i.e., number of concurrent harvesters, filtering out specific document types, required number of terms, etc.) For the acquisition of monolingual corpora, we used the focused crawler's monolingual mode of operation (FMC), which is also available as a web service.^9^

To initialise the crawler for the *env* domain, we constructed lists of seed URLs selected from relevant lists in the Open Directory Project.^10^ Alternative resources include the Yahoo^11^ directory. For the *lab* domain, similar lists were not so easy to find. The seed lists were therefore generated from queries for random combinations of terms using the WebBootCat toolkit (Baroni et al. 2006). When a page is fetched by the crawler, it is parsed in order to extract its metadata and content and normalised to the UTF-8 encoding. Next, the language is identified using the *n*-gram-based method included in the Apache Tika toolkit.^12^ In order to detect parts of text not in the targeted language, the language identifier is also applied at paragraph level and these parts are marked as such. The next processing step concerns boilerplate detection. For this task, we used a modified version of Boilerpipe (Kohlschütter et al. 2010), which also extracts structural information (such as title, heading, and list item), and segments text in paragraphs exploiting HTML tags. Paragraphs judged to be boilerplate are filtered out and each normalised page is then compared to the domain definition.

**Table 2:** Statistics from the trial and main phase of crawling of monolingual data: *pages stored* refers to the subset of *pages visited* and classified as in-domain, *pages deduped* refers to the pages after near-duplicate removal, *time* is total duration (in hours), and *acc* is accuracy estimated on the *pages sampled* that were crawled and classified during the trialphase

Language	Domain	Trial phase				Main phase						
		Sites	Pages all	Pages sampled	Acc (%)	Sites	Pages visited	Pages stored	$(\varDelta \%)$	Pages deduped	$(\varDelta \%)$	time (h)
English	*env*	146	505	224	*92.9*	3,181	90,240	34,572	*38.3*	28,071	*18.8*	47
	*lab*	150	461	215	*91.6*	1,614	121,895	22,281	*18.3*	15,197	*31.8*	50
French	*env*	106	543	232	*95.7*	2,016	160,059	35,488	*22.2*	23,514	*33.7*	67
	*lab*	64	839	268	*98.1*	1,404	186,748	45,660	*27.2*	26,675	*41.6*	72
Greek	*env*	112	524	227	*97.4*	1,104	113,737	31,524	*27.7*	16,073	*49.0*	48
	*lab*	117	481	219	*88.1*	660	97,847	19,474	*19.9*	7,124	*63.4*	38
Average					*94.0*				*25.6*		*39.7*	

The comparison to the domain definition is based on the amount of term occurrences, their location in the web page (i.e., title, keywords, body) and their weights. The page relevance score $p$ is calculated as proposed by Ardö and Golub (2007):

$$\begin{aligned} p = \sum \limits _{i=1}^N \sum \limits _{j=1}^4 n_{ij}\cdot w_{i}^t\cdot w_{j}^l, \end{aligned}$$

where $N$ is the amount of terms in the domain definition, $w_{i}^t$ is the weight of term $i$, $w_{j}^l$ is the weight of location $j$, and $n_{ij}$ denotes the number of occurrences of term $i$ in location $j$. The four discrete locations in a web page are *title*, *metadata*, *keywords*, and *html body*, with respective weights of 10, 4, 2, and 1 as proposed by Ardö and Golub (2007). If $p$ is higher than a predefined threshold, the web page is classified as relevant to the domain and stored. The threshold is the minimum number of terms to be found (the default value is 3) multiplied by the median value of the weights of all terms in the domain definition. It is worth mentioning that the user can favour precision over recall by setting the number of terms in the crawler’s configuration file. Similarly, the page relevance score to each subdomain is calculated and if this score is higher than the threshold, the web page is also classified as relevant to the corresponding subdomain(s). Otherwise the document is considered to be in the “unknown” subdomain.

Even when a page is not classified as relevant, it is still parsed and its links are extracted and added to the list of links to be visited. The fact that we keep links from non-relevant pages allows us to exploit the Tunnelling strategy (Bergmark et al. 2002), according to which the crawler does not give up examining a path when it encounters an irrelevant page. Instead, it continues searching that path for a predefined number of steps (the default value is 4), which allows the crawler to travel from one relevant web cluster to another when the number of irrelevant pages between them is beneath some threshold.

Although it is important to prevent the crawler from being ‘choked’, it is critical for crawl evolution to force the crawler to first follow links pointing to relevant pages. Therefore, we also adopted the Best-First algorithm in our implementation since this strategy is considered the baseline for almost all relevant related work. To this end, a link relevance score $l$ influenced by the source web page relevance score $p$ and the estimated relevance of the link’s surrounding text is calculated as

$$\begin{aligned} l = \frac{p}{N} + \sum _{i=1}^M n_{i}\cdot w_{i}, \end{aligned}$$

where $N$ is the amount of links originating from the source page, $M$ is the amount of terms in the domain definition, $n_{i}$ denotes the number of occurrences of the $i$-th term in the surrounding text and $w_{i}$ is the weight of the $i$-th term. This formulation of the link score was inspired by the conclusion of Cho et al. (1998), who stated that using a similarity metric that considers the content of anchors tends to produce some amount of differentiation between out-links and forces the crawler to visit relevant web pages earlier. New and unvisited links are merged and then sorted by their scores so that the most promising links are selected first for the next cycle. The statistics from the acquisition procedure are provided in Table 2.

**Fig. 1:**
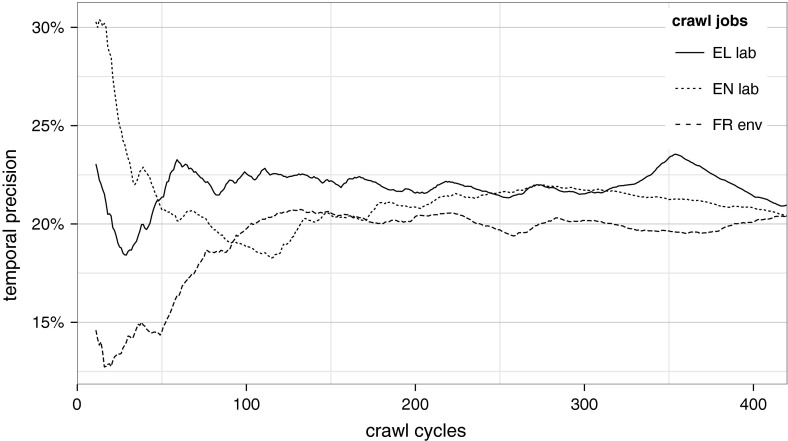
Visualisation of temporal precision (ratio of stored/visited pages per cycle) during three crawls

In order to estimate the crawler’s accuracy in acquiring in-domain resources, we first ran trial crawls in English, French, and Greek for the *env* and *lab* domains and asked native speakers to classify a sample of the acquired documents as domain-relevant or not based on provided domain descriptions (see Appendix). The results of the trial phase are given in columns 3–6 in Table 2). The average accuracy over all data sets is 94.0 % (see column 6).

Then we repeated the crawls to acquire larger collections (see columns 7–13). Duplicate web pages were detected and removed based on MD5 hashes, and near-duplicates were eliminated by employing the deduplication strategy implemented in the Nutch framework, which involves construction of a text profile based on quantised word frequencies.

As shown in column 10 of Table 2, the average precision at the end of the crawl jobs is about 25 %, a result similar to the conclusions reached by Srinivasan et al. (2005) and Dorado (2008). Figure 1 further illustrates the variation of the crawler’s temporal precision (i.e., the ratio of stored over visited pages after each crawling cycle) during the evolution of 3 crawls, where the average temporal precision remains above 20 % after 400 crawling cycles (the default value of the maximum number of URLs to be visited per cycle is 256).

The $\varDelta \hbox {s}$ of the 12th column in Table 2 refer to the percentage of documents removed during deduplication. The relatively high percentages of documents removed during deduplication is in accordance with the observation of Baroni et al. (2009), where during compilation of the Wacky corpora the amount of documents was reduced by more than 50 % following deduplication. Another observation is that the percentages of duplicates for the *lab* domain are much higher than the ones for *env* for all languages. This is explained by the fact that the web pages related to *lab* are often legal documents or press releases replicated on many websites.

The final processing of the monolingual data was performed on paragraphs marked by Boilerpipe and the language identifier. The statistics from this phase are presented in Table 3. Firstly, we discarded all paragraphs in languages different from the targeted ones as well as those classified as boilerplate, which reduced their total amount to 23.3 % on average. Removal of duplicate paragraphs then reduced the total number of paragraphs to 14 % on average. Most of the removed paragraphs, however, were very short chunks of text. In terms of tokens, the reduction is only 50.6 %. The last three columns in Table 3 refer to the final monolingual data sets used for training language models. For English and French, we acquired about 45 million tokens for each domain; for Greek, which is less frequent on the web, we obtained only about 15 and 20 million tokens for *lab* and *env*, respectively. These datasets are available from the ELRA catalogue^13^ under reference numbers ELRA-W00063–ELRA-W00068.

**Table 3:** Statistics from the cleaning stage of monolingual data acquisition and of the final data set: *paragraphs clean* refers to the paragraphs classified as non-boilerplate, and *paragraphs unique* to those obtained after duplicate removal

Language	Domain	Paragraphs all	Paragraphs cleaned	($\varDelta \%$)	Paragraphs unique	($\varDelta \%$)	Sentences	Tokens	Vocabulary
English	*env*	5,841,059	1,088,660	*18.6*	693,971	*11.9*	1,700,436	44,853,229	225,650
	*lab*	3,447,451	896,369	*26.0*	609,696	*17.7*	1,407,448	43,726,781	136,678
French	*env*	4,440,033	1,069,889	*24.1*	666,553	*15.0*	1,235,107	42,780,009	246,177
	*lab*	5,623,427	1,382,420	*24.6*	822,201	*14.6*	1,232,707	46,992,912	180,628
Greek	*env*	3,023,295	672,763	*22.3*	352,017	*11.6*	655,353	20,253,160	324,544
	*lab*	2,176,571	521,109	*23.9*	284,872	*13.1*	521,358	15,583,737	273,602
Average				*23.3*		*14.0*			

Statistics about the distribution of the subdomains of *env* and *lab* in English are presented in Table 4. The distributions for the Greek and French collections are similar, so we do not present them here. The main observation is that the collections are biased to specific subdomains. For example, “labour market” and “labour law and labour relations” cover 28.62 % and 25.68 % of the English *lab* data, respectively. This is due to the popularity of these subdomains in comparison with the rest, as well as the fact that the crawler’s goal was to acquire in-domain web pages without a requirement to build corpora balanced equally across subdomains. Another observation is that many documents were classified as parts of two subdomains. For example, 38.09 % of the documents in the English *env* collection were categorised in both “deterioration of the environment” and “natural environment”. This is explained by the fact that many terms of the domain definition were assigned to more than one subdomain. In addition, many crawled pages contain data relevant to these neighbouring subdomains.

**Table 4:** Distribution of subdomains in the monolingual English data crawled for the *env* and *lab* domains

	Ratio (%)
*Environment*	
Deterioration of the environment; natural environment	38.09
Natural environment	35.63
Environmental policy; natural environment	8.60
Energy policy	4.10
Deterioration of the environment; environmental policy; natural environment	3.34
Deterioration of the environment	2.64
Environmental policy	2.61
Cultivation of agricultural land	2.28
Deterioration of the environment; environmental policy	2.16
*Unknown*	0.56
Total	100.00
*Labour legislation*	
Labour market	28.62
Labour law and labour relations	25.68
Organisation of work and working conditions	12.46
Labour market; organisation of work and working conditions	6.76
Labour law and labour relations; labour market	5.46
Employment	4.26
Employment; labour market	3.59
Labour law and labour relations; organisation of work and working conditions	3.40
Personnel management and staff remuneration	3.05
Labour market; personnel management and staff remuneration	2.76
*Unknown*	2.07
Labour law and labour relations; labour market; organisation of work and working conditions	1.90
Total	100.00

### Acquisition of parallel texts

**Fig. 2:**
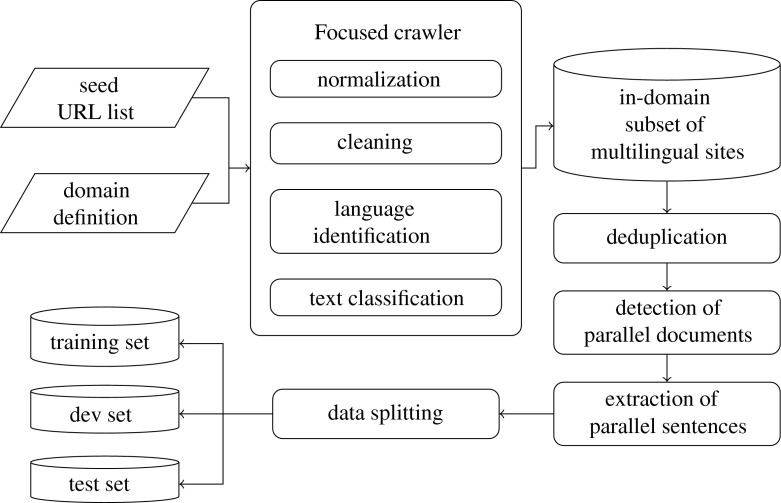
The entire workflow of parallel data acquisition resulting in training, development, and test sets

We now describe the procedure for acquisition of parallel data. To this end, we used the focused crawler's bilingual mode of operation (FBC), which is also available as a web service.^14^ Apart from the components for monolingual data acquisition (normalisation, language identification, cleaning, text classification and deduplication), this mode integrates a component for detection of parallel web pages, as illustrated in Fig. 2.

To guide FBC we used bilingual domain definitions, which consisted of the union of monolingual domain definitions in the targeted languages for the selected domain. In order to construct the list of seed URLs, we manually selected web pages that were collected during the monolingual crawls and originated from in-domain multilingual web sites. We then initialised the crawler with these URLs and forced the crawler to follow only links internal to these sites. By adopting the same crawling strategy mentioned in the previous subsection, FBC follows the most promising links and continues crawling the web site until no more internal links can be extracted.

After downloading in-domain pages from the selected web sites, we employed Bitextor to identify pairs of pages that could be considered translations of each other. Specifically, for each candidate pair of pages, we examine the relative difference in file size, the relative difference in length of plain text, the edit distance of web page fingerprints constructed on the basis of HTML tags, and the edit distance of the lists of numbers in the documents. If all measures are below corresponding thresholds, as those are defined in the default configuration of Bitextor, the pair under consideration is considered a pair of parallel pages. The amount of the acquired in-domain bilingual data is reported in columns 3 and 4 of Table 5.

### Extraction of parallel sentences

After identification of parallel pages, the next steps of the procedure aim at extraction of parallel sentences, i.e., sentence pairs that are likely to be mutual translations. For each document pair free of boilerplate paragraphs, we applied the following steps: identification of sentence boundaries by the Europarl sentence splitter, tokenisation by the Europarl tokeniser (Koehn 2005), and sentence alignment by Hunalign (Varga et al. 2005). Hunalign implements a heuristic, language-independent method for identification of parallel sentences in parallel texts, which can be improved by providing an external bilingual dictionary of word forms. If no such dictionary is provided, Hunalign builds it automatically from the data to be aligned. Without having such (external) dictionaries for EN–FR and EN–EL at hand, we obtained them by applying Hunalign to realign Europarl data in these languages. The resulting dictionaries were consequently used to improve sentence alignment of our in-domain data.

For each sentence pair identified as parallel, Hunalign provides a confidence score, which reflects the level of parallelism, i.e., the degree to which the sentences are mutual translations. We manually investigated a sample of sentence pairs extracted by Hunalign from the data pool for each domain and language pair (about 50 sentence pairs for each language pair and domain), by relying on the judgement of native speakers, and estimated that sentence pairs with a score above 0.4 are of sufficient translation quality. In the next step, we kept sentence pairs with 1:1 alignment only (one sentence on each side) and removed those with scores below this threshold. Finally, we also removed duplicate sentence pairs.

The statistics from the parallel data acquisition procedure are displayed in Table 5. An average of 84 % of source sentences extracted from the parallel documents were aligned 1:1, 10 % of these were then removed due to low estimated translation quality, and after discarding duplicate sentence pairs we ended up with 73 % of the original source sentences aligned to their target sides.

### Manual correction of test sentence pairs

The translation quality of a PB-SMT system built using the parallel sentences obtained by the procedure described above might not be optimal. Tuning the procedure and focusing on high-quality translations is possible, but leads to a trade-off between quality and quantity. For translation model training, high translation quality of the data is less essential than for tuning and testing. Bad phrase pairs can be removed from the SMT translation tables according, for example, to significance testing (Johnson et al. 2007). However, a development set containing sentence pairs that are not good translations of each other might lead to sub-optimal values of model weights, which would significantly harm system performance. If such sentences are used in the test set, the evaluation would be unreliable.

In order to create reliable test and development sets for each language pair and domain, we performed the following low-cost procedure. From the data obtained by the steps described in Sect. 3.3, we selected a random sample of 3,600 sentence pairs (2,700 for EN–EL in the *lab* domain, for which less data was available) and asked native speakers to check and correct them. All 4 evaluators (2 for each language) were researchers with postgraduate education and significant experience in evaluation for NLP tasks. The task consisted of checking that the sentence pairs belonged to the right domain, the sentences within a sentence pair were equivalent in terms of content, and the translation quality was adequate and if not, correcting it.

**Table 5:** Statistics from the parallel data acquisition procedure: websites used to crawl the data from (*sites*), total document pairs (*documents*), source-side sentences (*sentences all*), aligned sentence pairs (*paired*), those of sufficient translation quality (*good*); after duplicate removal (*unique*); sentences randomly selected for manual correction (*sampled*) and those manually validated and (if necessary) corrected (*corrected*); details in Table 6

Language pair	Domain	Sites	Documents	Sentences all	Sentences paired	($\varDelta \%$)	Sentences good	($\varDelta \%$)	Sentences unique	($\varDelta \%$)	Sentences sampled	Sentences corrected
English–French	*env*	6	559	19,042	14,881	*78.1*	14,079	*73.9*	13,840	*72.7*	3,600	3,392
	*lab*	4	900	35,870	31,541	*87.9*	27,601	*76.9*	23,861	*66.5*	3,600	3,411
English–Greek	*env*	14	288	17,033	14,846	*87.2*	14,028	*82.4*	13,253	*77.8*	3,600	3,000
	*lab*	7	203	13,169	11,006	*83.6*	9,904	*75.2*	9,764	*74.1*	2,700	2,506
Average						*84.2*		*77.1*		*72.8*

**Table 6:** Statistics (%) of manual correction of a sample of parallel sentences extracted by Hunalign

	EN–EL/*env*	EN–FR/*lab*
1. Perfect translation	53.49	72.23
2. Minor corrections done	34.15	21.99
3. Major corrections needed	3.00	0.33
4. Misaligned sentence pair	5.09	1.58
5. Wrong domain	4.28	3.86
Total	100.00	100.00

Our goal was to obtain at least 3,000 correct sentence pairs (2,000 test pairs and 1,000 development pairs) for each domain and language pair. Accordingly, in order to speed up the process, we did not instruct the correctors to amend every sentence pair, but rather allowed them to skip (remove) any sentence pairs that were misaligned. In addition, we asked them to remove those sentence pairs that were obviously from a very different domain (despite being correct translations). The number of manually verified and (if necessary) corrected sentence pairs is presented in the last column in Table 5.

According to the human judgements, 53–72 % of sentence pairs were accurate translations, 22–34 % needed only minor corrections, 1–3 % would require major corrections (which was not necessary, as the accurate sentence pairs together with those requiring minor corrections were enough to reach our goal of at least 3,000 sentence pairs), 2–5 % of sentence pairs were misaligned and would have had to be translated completely (which was not necessary in most cases), and about 4 % of sentence pairs were from a different domain (though correct translations). Detailed statistics collected during the corrections are presented in Table 6.

In the next step, we selected 2,000 pairs from the corrected sentences for the test set and left the remaining part for the development set. Those parallel sentences which were not sampled for the correction phase were added to the training sets. The correctors confirmed that the manual corrections were about 5–10 times faster than translating the sentences from scratch, so this can be viewed as a low-cost method for acquiring in-domain test and development sets for MT. Further statistics of all parallel data sets are given in Table 7. The data sets are available from ELRA under reference numbers ELRA-W0057 and ELRA-W0058.

**Table 7:** Statistics of the domain-specific parallel data sets obtained by web crawling and manual correction

Language pair (L1–L2)	Domain	Set	Corrected	Sentence pairs	L1 tokens	L1 vocabulary	L2 tokens	L2 vocabulary
English–French	*env*	Train	No	10,240	300,760	10,963	362,899	14,209
		Dev	Yes	1,392	41,382	4,660	49,657	5,542
		Dev^*raw*^	No	1,458	42,414	4,754	50,965	5,700
		Test	Yes	2,000	58,865	5,483	70,740	6,617
	*lab*	Train	No	20,261	709,893	12,746	836,634	17,139
		Dev	Yes	1,411	52,156	4,478	61,191	5,535
		Dev^*raw*^	No	1,498	54,024	4,706	63,519	5,832
		Test	Yes	2,000	71,688	5,277	84,397	6,630
English–Greek	*env*	Train	No	9,653	240,822	10,932	267,742	20,185
		Dev	Yes	1,000	27,865	3,586	30,510	5,467
		Dev^*raw*^	No	1,134	32,588	3,967	35,446	6,137
		Test	Yes	2,000	58,073	4,893	63,551	8,229
	*lab*	Train	No	7,064	233,145	7,136	244,396	14,456
		Dev	Yes	506	15,129	2,227	16,089	3,333
		Dev^*raw*^	No	547	17,027	2,386	18,172	3,620
		Test	Yes	2,000	62,953	4,022	66,770	7,056

## Baseline translation system

We now present our experimental set-up, the baseline (general-domain) system and its performance. Our primary evaluation measure is BLEU (Papineni et al. 2002) always reported as percentages. For detailed analysis, we also present PER (Tillmann et al. 1997) and TER (Snover et al. 2006) in Tables 17–20. The latter two are error rates, so the lower the score the better. In this paper, however, we report the scores as $(1-\hbox {PER})\times 100$ and $(1-\hbox {TER})\times 100$ respectively so that all metrics are in the range 0–100 where higher scores indicate better translations.

### System description

Our SMT system is MaTrEx (Penkale et al. 2010), a combination-based multi-engine architecture developed at Dublin City University. The architecture includes various individual systems: phrase-based, example-based, hierarchical phrase-based, and tree-based MT. In this work, we only exploit the phrase-based component, which is based on Moses (Koehn et al. 2007), an open-source toolkit for SMT.

For training, all data sets are tokenised and lowercased using the Europarl tools.^15^ The original (non-lowercased) target side of the parallel data is kept for training the Moses recaser. The lowercased versions of the target side are used for training an interpolated 5-gram language model with Kneser-Ney discounting (Kneser and Ney 1995) using the SRILM toolkit (Stolcke 2002). The parallel training data is lowercased and filtered at the sentence level; we kept all sentence pairs having fewer than 100 words on each side and with the length ratio within the interval $\langle 0.11,9.0\rangle$. The maximum length for aligned phrases is set to seven and the reordering models are generated using the parameters *distance* and *orientation-bidirectional-fe*. The resulting system combines the 14 feature functions described in Sect. 2.3.

The corresponding parameters are optimised on the development sets by MERT. After running several experiments with MERT, we found out that variance of BLEU caused by parameter optimization is quite low (about $\pm 0.25$ and in almost all cases statistically not significant) and due to the high number of experiments, for most systems we tune the parameters only once. In Sect. 5.2, we analyse the weights assigned by MERT to each parameter in our various experimental set-ups. For decoding, test sentences are also tokenised and lowercased. The evaluation measures are applied on tokenised and lowercased outputs and reference translations. To test statistical significance, we use paired bootstrap resampling for BLEU (Koehn 2004) with $p<0.05$ and 10,000 samples. In tables presenting the translation results in the following sections, the best scores for each translation direction and domain, and those which are statistically indistinguishable from the best ones are typed in bold.

### General-domain data

For the baseline general-domain system, we exploited the widely used data provided by the organisers of the SMT workshops (WPT 2005^16^ – WMT 2011^17^): the Europarl parallel corpus (Koehn 2005) as training data for translation and language models, and the WPT 2005 test sets as the development and test data for general-domain tuning and testing, respectively.

**Table 8:** Statistics of the general-domain data sets obtained from the Europarl corpus and the WPT workshop

Language pair (L1–L2)	Domain	Set	Source	Sentence pairs	L1 tokens	L1 vocabulary	L2 tokens	L2 vocabulary
English–French	*gen*	Train	Europarl 5	1,725,096	47,956,886	73,645	53,262,628	103,436
		Dev	WPT 2005	2,000	58,655	5,734	67,295	6,913
		Test	WPT 2005	2,000	57,951	5,649	66,200	6,876
English–Greek	*gen*	Train	Europarl 5	964,242	27,446,726	61,497	27,537,853	173,435
		Dev	WPT 2005	2,000	58,655	5,734	63,349	9,191
		Test	WPT 2005	2,000	57,951	5,649	62,332	9,037

Europarl is extracted from the proceedings of the European Parliament which covers a number of topics (Koehn 2005), including some related to the domains of our interest. For this reason, we take this corpus as a base for our domain-adaptation experiments and consider it to be general-domain. There is also a practical motivation for doing this: this corpus is relatively large, available for many language pairs, easily accessible for both industry and academia, and can be expected to play the same role in real-world applications. Europarl version 5, released in 2010, comprises texts in 11 European languages including all languages of interest in this work (see Table 8). Note that the amount of parallel data for EN–EL is only about half of what is available for EN–FR. Furthermore, Greek morphology is more complex than French morphology so the Greek vocabulary size (we count unique lowercased alphabetical tokens) is much larger than the French one. The WPT 2005 development and test sets contain 2,000 sentence pairs each, available in the same languages as Europarl provided by the WPT 2005 organisers as development and test sets for the translation shared task (later WMT test sets do not include Greek data). All data sets used in our experiments contain a single reference translation.

### Baseline system evaluation

A number of previously published experiments (e.g., Wu et al. 2008; Banerjee et al. 2010) reported significant degradation in translation quality when an SMT system was applied to out-of-domain data. In order to verify this observation, we compare the performance of the baseline system (trained and tuned on general-domain data) on all our test sets: general-domain (*gen*) and domain-specific (*env*, *lab*). We present the results in Table 9.

**Table 9:** Performance comparison of the baseline systems (*B0*) tested on general (*gen*) and specific (*env*, *lab*) domains

Direction	General			Environment				Labour legislation			
	BLEU	OOV	PPL	BLEU	$\varDelta$	OOV	PPL	BLEU	$\varDelta$	OOV	PPL
English–French	52.57	0.11	28.1	29.61	$-$22.96	0.98	67.8	23.94	$-$28.63	0.85	83.2
French–English	57.16	0.11	32.0	31.79	$-$25.37	0.81	122.0	26.96	$-$30.20	0.68	153.6
English–Greek	42.52	0.22	130.0	21.20	$-$21.32	1.15	119.8	24.04	$-$18.48	0.47	82.1
Greek–English	44.30	0.56	36.0	29.31	$-$14.99	1.53	115.4	31.73	$-$12.57	0.69	74.9
Average		0.25	56.6		$-$21.16	1.12	106.4		$-$22.47	0.67	98.5

$\varDelta$ refers to the change in BLEU score over the *gen* domain, OOV to the out-of-vocabulary rate (%) of the test sentences, and PPL to perplexity of the reference translations given the target-side language models

The BLEU scores obtained on the general domain test sets are quite high—they range from 42.52 to 57.16 points. This is caused by the fact that the development and test sentence pairs were taken from the same source (proceedings of the European Parliament), where similar expressions and phrases often recur. We found that about 5 % of EN–FR development and test sentence pairs also occur in the training data (although no sentence pair duplicates were found in the EN–EL test sets). The duplicates were probably added to later versions of Europarl after the WPT 2005 test sets were released, but this does not affect our domain-adaptation experiments presented in this paper.

Switching from general-domain to domain-specific test sets yields an average absolute decrease of 21.16 BLEU points (48.22 % relative) on the *env* domain and 22.47 BLEU points (44.84 % relative) on the *lab* domain (see columns denoted by $\varDelta$ in Table 9). Although the magnitude of the decrease might be a little overestimated (due to the occurrence of a portion of the *gen* test data in the training data), the drop in translation quality is evident. It is caused by the divergence of training and test data, which is also illustrated by the increase of the OOV rates (ratios of untranslated words) and perplexity (PPL) of the reference translations of the test sets given language models trained on the target side of the parallel training data (this reflects how well the language model reflects the characteristics of the target language). For both measures, lower scores indicate a better fit.

The OOV rate increases from an average of 0.25 % on the *gen* domain to 1.12 % on the *env* domain and 0.67 % on the *lab* domain, and the average perplexity increased from 56.6 on the *gen* domain to 106.4 on the *env* domain and 98.5 on the *lab* domain (see Table 9). It almost doubles when going from general (*gen*) to specific (*env*, *lab*) domain and makes scoring of hypotheses during decoding difficult. An interesting case is the EN–EL translation direction, where the highest perplexity is surprisingly achieved on the *gen* domain. This is probably due to the morphological complexity of the target language and nature of the particular test set. After a thorough analysis of the Greek side of this test set, we discovered some inconsistency in tokenisation (introduced by the providers of the data) which contributed to the higher PPL value. This does not, however, influence the findings in this work. In all other cases, perplexity increases for domain-specific data.

## Domain adaptation by parameter tuning

Optimisation of the log-linear combination parameters, which most modern SMT systems are based on, is known to have a big influence on translation performance. A sensible first step towards domain adaptation of a general-domain system is to use in-domain development data. Such data usually comprises a small set of parallel sentences which are repeatedly translated until the model parameters are adjusted to their optimal values.^18^

**Table 10:** Parameter tuning of the baseline (general-domain-trained) systems on various development data: general-domain (*B0*), corrected in-domain sentences (*P1*), raw in-domain sentences (*P2*), cross-domain data (*P3*), and by using the default weights (*P4*)

Direction	Test	General (*B0*)	In-domain (*P1*)		In-domain^raw^ (*P2*)		Cross-domain (*P3*)		Default (*P4*)	
		BLEU	BLEU	$\varDelta$	BLEU	$\varDelta$	BLEU	$\varDelta$	BLEU	$\varDelta$
English–French	*env*	29.61	**37.51**	**7.90**	37.25	7.64	**37.47**	**7.86**	36.24	6.63
	*lab*	23.94	**32.15**	**8.21**	31.88	7.94	31.82	7.88	30.60	6.66
French–English	*env*	31.79	**39.05**	**7.26**	**38.93**	**7.14**	**38.79**	**7.00**	34.05	2.26
	*lab*	26.96	**33.48**	**6.52**	**33.34**	**6.38**	33.07	6.11	29.69	2.73
English–Greek	*env*	21.20	**27.56**	**6.36**	27.29	6.09	27.26	6.06	27.16	5.96
	*lab*	24.04	**30.07**	**6.03**	**30.23**	**6.19**	29.68	5.64	29.76	5.72
Greek–English	*env*	29.31	**34.31**	**5.00**	**34.32**	**5.01**	33.98	4.67	31.45	2.14
	*lab*	31.73	**37.57**	**5.84**	**37.68**	**5.95**	**37.58**	**5.85**	34.95	3.22
Average				6.64		6.54		6.38		4.42

$\varDelta$ refers to absolute improvement in BLEU over the baseline (*B0*)

By using the parallel data acquisition procedure described in Sect. 3, we acquired development sets (506–1,411 sentence pairs depending on the language pair), which prove to be very beneficial for parameter tuning in our experiments (see Table 10). Compared to the baseline systems trained and tuned on general-domain data only (denoted as *B0)*, the systems trained on general-domain data and tuned on in-domain data (denoted as *P1*) improve BLEU by 6.64 points absolute (24.82 % relative) on average (compare columns *B0* and *P1* in Table 10). On the one hand, this behaviour is to be expected, but taking into account that the development sets contain only several hundreds of parallel sentences each, such an improvement is nevertheless significant.

### Correction of development data

A small amount of manual effort was put into the manual correction of the test as well as development data acquired for the specific domains (see Sect. 3.4). In order to assess the practical need to correct the development data, we compare baseline systems tuned on manually corrected development sets with systems tuned on raw development sets. This raw development data (denoted by ${raw}$ in Table 7) contains not only the sentences with imperfect translation, but also those that are misaligned and/or belong to other domains. As a consequence, the raw development sets contain 5–14 % more sentence pairs than the corrected ones (see Table 7). The performance of the systems tuned using the raw development data is shown in Table 10, column *P2*. In general, the absolute differences in BLEU compared to the *P1* systems are very small and not statistically significant for most of the scenarios (figures in bold). The average absolute improvement over the baseline system *B0* is 6.54 BLEU points, which is only 0.1 points less than the score obtained by the *P1* systems. In practice, this finding makes the manual correction of development data acquired by our procedure unnecessary since the results obtained using raw parallel data are comparable.

### Analysis of model parameters

**Fig. 3:**
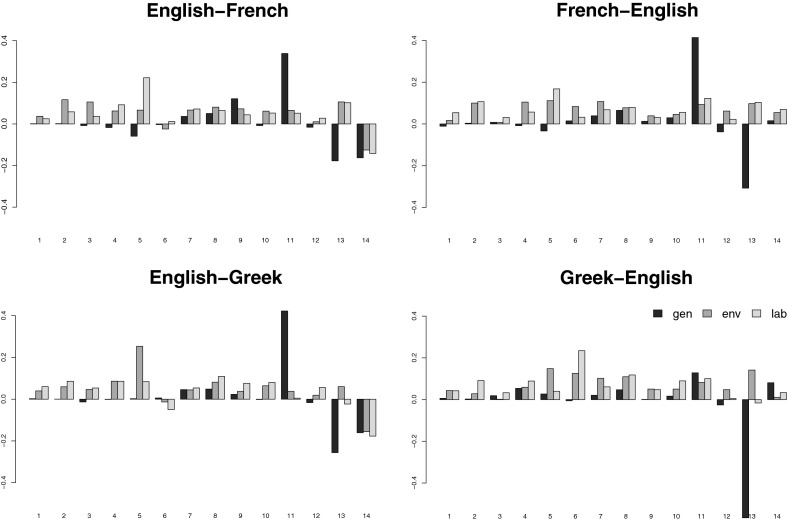
Visualisation of model weights of the four systems in the twelve evaluation scenarios; the *black bars* refer to model weights of the systems tuned on general-domain (*gen*) development sets, while the *grey bars* refer to the model weights of the systems tuned on domain-specific development sets (*env*, *lab*)

The only things that change when the systems are tuned on in-domain data are the weights of the feature functions in the log-linear combination optimised by MERT. The reordering, language, and translation models all remain untouched, as they are trained on general-domain data. Recall that the parameter space searched through by MERT is large and the error surface highly non-convex, so the resulting weight vectors might not be globally optimal and there might be other (i.e., different) weight vectors that perform equally well or even better. For this reason, the actual parameter values are not usually investigated. Our experiments, however, show that the parameter values and their changes observed when switching from general-domain to domain-specific tuning are in fact highly consistent, indicating interesting trends (compare the black and grey bars in Fig. 3).

The high weights assigned to $h_{11}$ (*direct phrase translation probability*) of the general-domain tuned systems (black bars) indicate that the phrase pairs in the systems’ translation tables apply well to the development data that are from the same domain as the training data; a high reward is given to translation hypotheses consisting of phrases with high translation probability (i.e., good general-domain translations). The low negative weights assigned to $h_{13}$ (*phrase penalty*) imply that the systems prefer hypotheses consisting of fewer but longer phrases. Reordering in the hypotheses is not rewarded and therefore not explicitly preferred (the weights of the reordering models $h_{1}$–$h_{7}$ are assigned values around zero). In some scenarios (e.g., for EN–FR and FR–EN), certain reordering schemes are even slightly penalised (several weights of $h_{1}$–$h_{7}$ have negative values). The weight of $h_{14}$ (*word penalty*) is negative for the systems translating from English and slightly positive for systems translating into English. This reflects the fact that translation from English prefers shorter hypotheses (fewer words), while translation into English prefers longer hypotheses (consisting of more words). This is probably due to the relative morphological complexities of English and the other languages.

Comparing these findings with the results of the systems tuned on the specific domains (grey bars), we observe that the weights of $h_{11}$ (*direct phrase translation probability*) decrease rapidly, with this weight being close to zero in some scenarios. The translation tables do not provide enough good quality translations for the specific domains, and the best translations of the development sentences consist of phrases with varying translation probabilities. Hypotheses consisting of few (and long) phrases are not rewarded any more (weights of $h_{13}$ are higher); in most cases they are penalised and hypotheses consisting of more (and short) phrases are allowed or even preferred. In almost all cases, the reordering feature weights (features $h_1$–$h_7$) increased substantially, and for domain-specific data the model significantly prefers hypotheses with specific reordering (which is consistent with the two preceding observations). Language model weights ($h_8$) do not change substantially, with its importance as a feature remaining similar on general-domain and domain-specific data.

As can be seen in Fig. 3, these findings are consistent across domains and language pairs. The weight vectors of the systems tuned on domain-specific data are quite similar but differ substantially from the parameters obtained by tuning on general domain data.

### Analysis of phrase-length distribution

From the analysis presented above, we conclude that a PB-SMT system tuned on data from the same domain as the training data strongly prefers to construct translations consisting of long phrases. Such phrases are usually of good translation quality (local mistakes of word alignment disappear), fluent (formed by consecutive sequences of words), and recurrent (frequent in data from the same domain). Accordingly, they form good translations of the input sentences and are preferred during decoding. This is, of course, a positive trait when the system translates sentences from the same domain. However, if this is not the case and the input sentences contain very few longer phrases from the translation tables, the general-domain tuned system is not able to construct good translations by preferring the longer and (for this domain) inadequate phrases. In this case, shorter phrases could enable better translations to be stitched together.

**Fig. 4:**
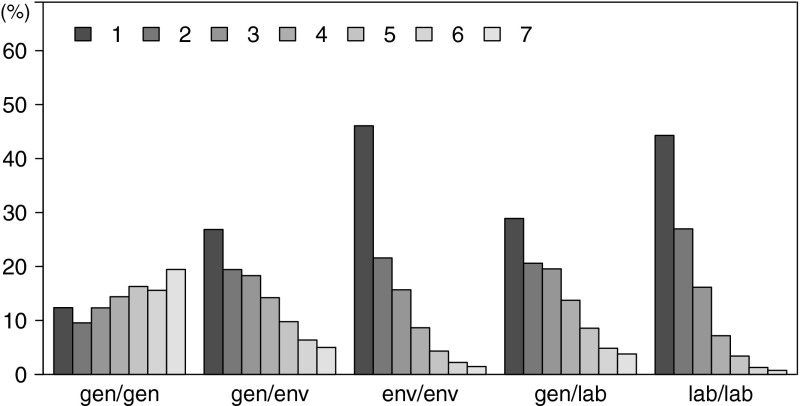
Distribution of phrase length in English–French translations by systems tuned/tested on various combinations of general (*gen*) and specific (*env*, *lab*) domains (maximum phrase length set to seven)

To support this hypothesis, we analysed the phrase length distribution actually seen in the translation of the test sets. The average phrase lengths estimated for various combinations of tuning and test domains and all language pairs are shown in Table 11. The highest values are observed for translations of general-domain test sets by systems tuned on the same domain: 3.49 on average across all language pairs. The scores for systems trained on general-domain and tuned and tested on domain-specific data are significantly lower and range from 1.54 to 2.97, depending on the domain and language pair. Figure 4 illustrates the complete phrase-length distribution in EN–FR translations by systems tuned and tested on various combinations of general and specific domains.

**Table 11:** Average phrase length in translations by systems tuned/tested on various combination of domains

	*gen*/*gen*	*gen*/*env*	*env*/*env*	*gen*/*lab*	*lab*/*lab*
English–French	4.37	3.00	2.16	2.82	2.05
French–English	3.46	2.49	1.77	2.45	1.83
English–Greek	3.76	2.69	2.17	2.97	2.46
Greek–English	2.35	2.18	1.54	2.43	2.30
Average	3.49	2.59	1.91	2.67	2.16

Generally, a higher divergence of the test domain from the training domain leads to shorter phrases being used in translation. However, when the systems tuned on general-domain data are applied to specific domains, the average phrase lengths are consistently longer than for domain-specific tuning. The systems are tuned to prefer long phrases but the translation quality is lower. This situation can be interpreted as overtraining; the model overfits the training (and tuning) data and on a different domain fails to form the best possible translations (given the translation, reordering, and language models). Nevertheless, preferring translations constructed of shorter phrases (even single words) is not always better. For example, word-by-word translation of non-compositional phrases would generally be erroneous.

### Other alternatives to parameter optimisation

As we have already shown, in-domain tuning represents a way to effectively reduce such overfitting. The problem, however, can also be reduced by cross tuning, i.e., tuning on specific domains different from the test domains (tuning on *lab* and testing on *env*, and vice versa), see Table 10, column *P3*. In three scenarios (bold figures), such systems perform as well as the in-domain tuned ones (no statistically significant difference). In the other scenarios, the absolute difference in BLEU is less than 0.4 points. The average gain over the systems tuned on the general domain (*B0*) is 6.38 points absolute (compared with 6.64 points obtained by *P1*). This observation is not very intuitive. One would expect that each domain would require specific tuning. However, it seems that the in-domain tuning does not optimize the general-domain trained system to a particular specific domain, but rather to *any* domain diverging from the general domain in a similar way (e.g., to the extent that the translation model and language model cover the test data).

For comparison purposes only, we also report results of non-tuned systems *P4* using the default weight vectors set by Moses ($h_{1,\dots ,7}=0.3, h_8=0.5, h_{9,\dots ,13}=0.2, h_{14}=-1$). Even this approach outperforms the baseline systems *B0*. In some cases (e.g., the EN–EL translations), the results are very close to those of systems tuned on in-domain data (*P1*). The average absolute improvement of the systems with default parameters (*P4*) over the systems tuned on general domain is 4.42 BLEU points (compared with 6.64 points obtained from domain-specific tuning on average).

### Analysis of learning curves

Often, domain-specific parallel data is scarce, or completely unavailable for many vertical sectors and must be prepared by manual translation of monolingual in-domain sentences. We thus investigate how much development data is needed. The only technical requirement is that the parameter optimisation method (MERT, here) must converge in a reasonable number of iterations. For this reason, typical development sets contain about 1,000–2,000 sentence pairs (cf. the size of development sets provided for the WMT translation shared tasks). We vary the amount of sentences in our development sets, tune the systems, test their performance on the test sets and plot learning curves to capture the correlation between translation quality (in terms of BLEU) and gradual increases in the size of the development data.

**Fig. 5:**
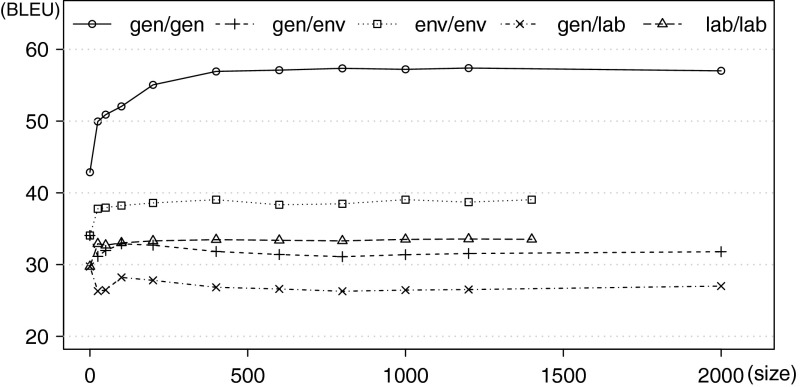
Translation quality (BLEU) of FR–EN systems tuned on data of varying size. The domains of the development and test sets are given in this order (*dev*/*test*)

The general shapes of the curves are consistent across all language pairs and thus we provide the curves for the EN–FR translation direction only (see Fig. 5). Increasing the size of development sets is beneficial only where the domains of development and test data are the same. The curve of the system tuned and tested on the general domain reaches a plateau at about 500 sentence pairs. In the case of in-domain tuning for specific domains, the plateau is reached much earlier. Usually, as few as 100–200 sentence pairs are enough to obtain optimal results. This is encouraging, as tuning on specific domains yields the best results, and fortunately requires only very limited amounts of bilingual data and seems reasonably tolerant to imperfect translations and noise in the development sentences. The development sets of more than 400–600 sentence pairs do not improve translation quality at all and make the tuning process take longer; by the same token, the additional tuning data does not actively degrade performance, so there is no need to reduce the size of the tuning set. The systems tuned on the general-domain data and tested on specific domains do not benefit from the development data at all; the initial and relatively high BLEU scores achieved with zero-size development data sets (i.e., no tuning) decrease with increasing size of the domain-specific development sets (see the curves denoted as *gen*/*env* and *gen*/*lab* in Fig. 5).

## Adaptation of language and translation models

In this section, we explore the potential of adapting the components of the SMT model (language and translation models) by exploiting the crawled domain-specific data in addition to the general-domain data used for training the baseline systems.

### Language model adaptation

Improving an SMT system by adding in-domain monolingual training data cannot reduce OOV rates nor introduce new phrase pairs into the translation models. Such data can, however, improve the language models and contribute to better estimates of translation fluency and thus help select better translation hypotheses.

In general, there are two ways of using monolingual data for adaptation of the SMT model: the trivial approach is to retrain the existing language model on a simple concatenation of the original general-domain data and the new domain-specific data; a more advanced approach is to build an additional language model based on the domain-specific data only and use it together with the original one. This is possible in two ways (Foster and Kuhn 2007): the two models can be merged by linear interpolation into one model or used directly as components in the log-linear combination of the system. The two approaches are similar but not identical. Both are parametrised by a single weight corresponding to the relative importance of the two models (a linear interpolation coefficient and a model weight, respectively) and require optimisation. Linear interpolation can be optimised by minimising perplexity of some target-language data (e.g., the target side of the development set). Log-linear combination allows direct optimisation of MT quality (e.g., by MERT).

**Table 12:** Results of language model adaptation by concatenation of training data (*L1*), linear interpolation of general-domain and domain-specific models (*L2*), and employing the two independent models in log-linear combination (*L3*)

Direction	Test	Base (*P1*)	Concatenation (*L1*)		Lin. interpol. (*L2*)		Log-lin. comb. (*L3*)	
		BLEU	BLEU	$\varDelta$	BLEU	$\varDelta$	BLEU	$\varDelta$
English–French	*env*	37.51	41.28	3.77	**41.78**	**4.27**	41.25	3.74
	*lab*	32.15	36.15	4.00	**38.54**	**6.39**	35.54	3.39
French–English	*env*	39.05	40.58	1.53	**42.63**	**3.58**	39.93	0.88
	*lab*	33.48	38.05	4.57	**41.11**	**7.63**	33.95	0.47
English–Greek	*env*	27.56	33.59	6.03	**34.89**	**7.33**	33.65	6.09
	*lab*	30.07	**35.09**	**5.02**	34.15	4.08	34.33	4.26
Greek–English	*env*	34.31	37.03	2.72	**37.57**	**3.26**	36.55	2.24
	*lab*	37.57	**40.15**	**2.58**	**40.09**	**2.52**	**40.01**	**2.44**
Average				3.78		4.88		2.94

$\varDelta$ refers to absolute improvement in BLEU over *P1* trained on general domain and tuned for specific domains

We experiment with all three approaches combining general-domain data (comprising 27–53 million tokens, see Table 8) and in-domain data (15–45 million tokens, see Table 3). System *L1* exploits the simple concatenation of the data, *L2* is based on linear combination optimized on the target side of the (in-domain) development data, and *L3* employs two models combined in the log-linear fashion using weights tuned by MERT on BLEU. The complete results are presented in Table 12. Compared to the in-domain tuned systems (*P1*), all three methods significantly improve translation quality across all scenarios. In general, the most efficient approach is linear interpolation with an average absolute improvement of 4.88 BLEU points (14.95 % relative). With two exceptions, systems *L2* outperform both *L1* and *L3*. In most cases, the improvement is statistically significant. For EN–EL (both directions) in the *lab* domain, *L2* is outperformed by simple concatenation (*L1*), but this can be explained by the size of the development data used to optimize the interpolation coefficient in *L2* (506 sentences), which is probably insufficient. Substantial improvements in BLEU over the system *P1* are achieved especially for translations into Greek (7.33 points for *env*, and 5.02 points for *lab*, both absolute) despite the smallest size of the monolingual data acquired for this language (see Table 3), which is probably due to the complex Greek morphology and the subsequent problem of data sparsity.

### Translation model adaptation

Parallel data is essential for building translation models of SMT systems. While a good language model can improve an SMT system by preferring better phrase translation options in given contexts, it has no effect if the translation model fails to provide a phrase translation at all. In this experiment, we analyse the effect of using the domain-specific parallel training data acquired as described in Sect. 3.2. These data sets are relatively small, comprising 7,000–20,000 sentence pairs, depending on the language pair and domain (see Table 5).

Similar to language model adaptation discussed in the previous subsection, there are three main methods to combine parallel training data from two sources (Banerjee et al. 2011): first, retraining the existing translation model on a simple concatenation of the original general-domain and the new domain-specific data; second, training a new translation model on the domain-specific data and interpolating the two models in a linear fashion; and third, using the two translation models in log-linear combination. The first approach does not require optimization of any additional parameters. The second approach requires tuning of four extra coefficients (one for each of the probability distributions provided by the translation model, i.e., $h_9$–$h_{12}$), which is usually done by minimizing perplexity of the development data (Sennrich 2012). The third approach adds the total of five new weights (associated with the new translation model) to the weight vector, which is then optimized in the traditional way by maximising translation quality on the development data (by MERT, in our case).

**Table 13:** Results of translation model adaptation by concatenation of training data (*T1*), linear interpolation of general-domain and domain-specific models (*T2*), and employing the independent models in log-linear combination (*T3*)

Direction	Test	Base (*P1*)	Concatenation (*T1*)		Lin. interpol. (*T2*)		Log-lin. comb. (*T3*)	
		BLEU	BLEU	$\varDelta$	BLEU	$\varDelta$	BLEU	$\varDelta$
English–French	*env*	37.51	**39.61**	**2.10**	**39.85**	**2.34**	**39.76**	**2.25**
	*lab*	32.15	41.33	9.18	42.08	9.93	**42.70**	**10.55**
French–English	*env*	39.05	41.08	2.03	**41.92**	**2.87**	41.65	2.60
	*lab*	33.48	43.54	10.06	**45.06**	**11.58**	**45.12**	**11.64**
English–Greek	*env*	27.56	30.73	3.17	30.74	3.18	**31.89**	**4.33**
	*lab*	30.07	**30.48**	**0.41**	**30.51**	**0.44**	**30.51**	**0.44**
Greek–English	*env*	34.31	38.35	4.04	38.12	3.81	**38.66**	**4.35**
	*lab*	37.57	**38.07**	**0.50**	**38.20**	**0.63**	37.90	0.33
Average				3.94		4.35		4.56

$\varDelta$ refers to absolute improvement in BLEU over *P1* trained on general domain and tuned for specific domains

We test all the alternative approaches, which are realised as systems *T1* (single translation model trained on a concatenation of data), *T2* (linear interpolation of the two translation models), *T3* (two independent translation models in log-linear combination), and compared with the in-domain-tuned systems (*P1*) in Table 13. We again observe substantial improvements in translation quality in all scenarios. However, there is no clear winner in this case: although the two more advanced methods (systems *T2* and *T3*) outperform the trivial one (system *T0*), the difference between the two is marginal. The average increase in BLEU for *T2* over *T1* is 4.35 points absolute (13.11 % relative) and for *T3* over *P1* 4.56 points absolute (13.87 % relative). In three of the eight scenarios, the difference is not statistically significant, *T2* is significantly better in two scenarios, and *T3* is better in three scenarios (see Table 13).

The most substantial gain obtained by exploiting the domain-specific parallel training data is observed for the EN–FR language pair (in both translation directions) and the *lab* domain, where BLEU scores increase by 10.55–11.64 points absolute (for system *T3*), while in other scenarios the increase in BLEU is between 0.33 and 4.35 points absolute only. This can be explained by the better match between the training and test data, which is evident from the decrease in perplexity of the reference translations given the target language models, as discussed in the following section. This is likely to be caused by the size of the in-domain parallel training data for this language pair and domain which is more than twice as large compared to the EN–FR *env* data and more than three times larger compared to the EN–EL data, both for the *env* and *lab* domains (see Table 3).

In further experiments, we test the techniques for translation model adaptation in systems with language models adapted by linear interpolation, which proved to be the most effective method for language model adaptation. Overall, the results presented in Table 14 are very positive: the improvements obtained by translation model adaptation are to a large extent preserved even when this method is applied together with language model adaptation. While linear interpolation of translation models realised in systems *T2* increases BLEU by 4.35 points absolute (*T2* over *P1*, see Table 13), the same technique adds an additional 3.78 BLEU points when applied together with linear interpolation of language models (*C2* over *L2*, see Table 14). The effect of using in-domain monolingual and parallel data is largely independent and does not cancel out when these two types of resources are used at the same time. On average, linear-interpolation outperforms the other two techniques (*C1* and *C3*), but in most scenarios the difference is not statistically significant (cf. the bold figures in Table 14).

**Table 14:** Results of complete adaptation. Language models in all systems are adapted by linear interpolation; translation models are adapted by concatenation of training data (*C1*), linear interpolation of general-domain and domain-specific models (*C2*), and employing the independent models in log-linear combination (*C3*)

Direction	Test	Base (*L2*)	Concatenation (*C1*)		Lin. interpol. (*C2*)		Log-lin. comb. (*C3*)	
		BLEU	BLEU	$\varDelta$	BLEU	$\varDelta$	BLEU	$\varDelta$
English–French	*env*	41.78	**43.70**	**1.92**	**43.85**	**2.07**	**43.75**	**1.97**
	*lab*	38.54	47.45	8.91	**48.31**	**9.77**	47.96	9.42
French–English	*env*	42.63	43.93	1.30	**44.22**	**1.59**	**44.12**	**1.49**
	*lab*	41.11	50.07	8.96	**50.56**	**9.45**	**50.34**	**9.23**
English–Greek	*env*	34.89	**38.41**	**3.52**	37.90	3.01	**38.22**	**3.33**
	*lab*	34.15	34.29	0.14	**34.76**	**0.61**	**34.48**	**0.33**
Greek–English	*env*	37.57	**40.85**	**3.28**	**40.64**	**3.07**	**40.81**	**3.24**
	*lab*	40.09	**40.69**	**0.60**	**40.75**	**0.66**	**40.62**	**0.53**
Average				3.58		3.78		3.69

$\varDelta$ refers to absolute improvement in BLEU over *L2* with translation models trained on general-domain data only

### Complete adaptation and result analysis

In this section, we summarise the main results achieved by incremental improvements obtained by adaptation of various components of a PB-SMT system. and compare them with the original baseline systems trained and tuned on general-domain data only. The results are accompanied by further analysis of three factors: OOV rate in test sentences, perplexity of reference translations given the target language models, and average phrase length in test translations.

The main results in terms of BLEU are presented in Table 15, with the detailed characteristics of the systems given in Table 16. On average, in-domain parameter tuning (*P1*) improves BLEU by 6.64 points absolute (24.82 % relative). Components of the log-linear combination do not change, so OOV and perplexity remain the same. The average phrase length dropped from 2.63 to 2.04 words, i.e., by 22.5 %. The adapted language model (log-linear interpolation of general-domain and domain-specific models tuned on the target side of development data, systems *L2*) increased the gain in BLEU to 11.52 points absolute (43.73 % relative). The perplexity of the reference translations given the target language models dropped by 45.4 % on average. The average phrase length decreased to 1.87 words. The language model matches the test data domain better and helps to select better translation hypotheses, which consist of even more (and shorter, eventually reordered) phrases.

Finally, adaptation of the translation model (using linear interpolation general-domain and domain-specific models tuned on the development data, systems *C2*) boosts the average relative improvement in BLEU to 15.30 points absolute (58.37 % relative). This step introduces new translation phrase pairs into the translation model and decreases the OOV rate. Compared to the baseline (*B0*), OOV drops by 30 % on average. In some scenarios (the EN–FR translation in the *lab* domain), OOV decreases by as much as 50 %, which is a sign of a better match between the test and training data. The target side of the parallel data also improves the language models, with their perplexity falling by an average of 67.5 % relative. The new in-domain material in the translation models also leads to longer phrases being used in the best-scored translation hypotheses. The average phrase length increased compared to the systems with adapted language models only (*L2*) by almost 20 % to 2.18 words.

**Table 15:** Incremental adaptation using various types of domain-specific resources: parallel data for parameter tuning (*P1*), monolingual data for improving the language models (*L2*), and parallel data for improving the translation model (*C2*)

Direction	Test	Base (*B0*)	+Tuning (*P1*)		+Lang. model (*L2*)		+Transl. model (*C2*)		Spec. only (*C0*)	
		BLEU	BLEU	$\varDelta$	BLEU	$\varDelta$	BLEU	$\varDelta$	BLEU	$\varDelta$
English–French	*env*	29.61	37.51	7.90	41.78	12.17	39.85	**14.24**	39.54	9.93
	*lab*	23.94	32.15	8.21	38.54	14.60	42.08	**24.37**	43.05	19.11
French–English	*env*	31.79	39.05	7.26	42.63	10.84	41.92	**12.43**	37.86	6.07
	*lab*	26.96	33.48	6.52	41.11	14.15	45.06	**23.60**	43.74	16.78
English–Greek	*env*	21.20	27.56	6.36	34.89	13.69	30.74	**16.70**	29.84	8.64
	*lab*	24.04	30.07	6.03	34.15	10.11	30.51	**10.72**	26.19	2.15
Greek–English	*env*	29.31	34.31	5.00	37.57	8.26	38.12	**11.33**	30.71	1.40
	*lab*	31.73	37.57	5.84	40.09	8.36	38.20	**9.02**	29.54	−2.19
Average				6.64		11.52		15.30		7.74

$\varDelta$ refers to absolute improvement in BLEU over the baseline general-domain system (*B0*)

**Table 16:** Out-of-vocabulary rate (%) in the test sentences (OOV), perplexity of the reference translations given the target language models (PPL), and average phrase length in the test set translations (APL)

Direction	Test	Base (*B0*)			+Tuning (*P1*)			+Lang. model (*L2*)			+Transl. model (*C2*)		
		OOV	PPL	APL	OOV	PPL	APL	OOV	PPL	APL	OOV	PPL	APL
English–French	*env*	0.98	67.8	3.00	0.98	67.8	2.16	0.98	36.7	2.18	0.65	33.3	2.60
	*lab*	0.85	83.2	2.82	0.85	83.2	2.05	0.85	40.9	1.91	0.48	29.2	2.70
French–English	*env*	0.81	122.9	2.49	0.81	122.9	1.77	0.81	80.9	1.75	0.54	68.3	2.15
	*lab*	0.68	153.6	2.45	0.68	153.6	1.83	0.68	59.5	1.54	0.38	40.2	1.81
English–Greek	*env*	1.15	119.7	2.69	1.15	119.7	2.17	1.15	50.6	1.85	0.82	43.8	2.09
	*lab*	0.47	82.1	2.97	0.47	82.1	2.46	0.47	50.4	1.86	0.40	49.1	2.10
Greek–English	*env*	1.53	115.4	2.18	1.53	115.4	1.54	1.53	76.3	1.66	1.20	72.2	2.25
	*lab*	0.69	74.9	2.43	0.69	74.9	2.30	0.69	53.2	2.16	0.62	52.5	1.78
Average		0.90	102.5	2.63	0.90	102.5	2.04	0.90	56.0	1.87	0.64	48.6	2.18

For comparison, Table 15 also reports the results of systems trained and tuned solely on domain-specific data (*C0*), which illustrates the pure effect of such training data. Although with one exception (EN–EL translation of the *lab* domain), these systems outperform the baseline (*B0*), the requirement of using general-domain data is evident in all scenarios. The average difference in BLEU of the fully adapted systems (*C2*) and the systems trained on specific data only (*C0*) is 7.56 points absolute.

**Fig. 6:**
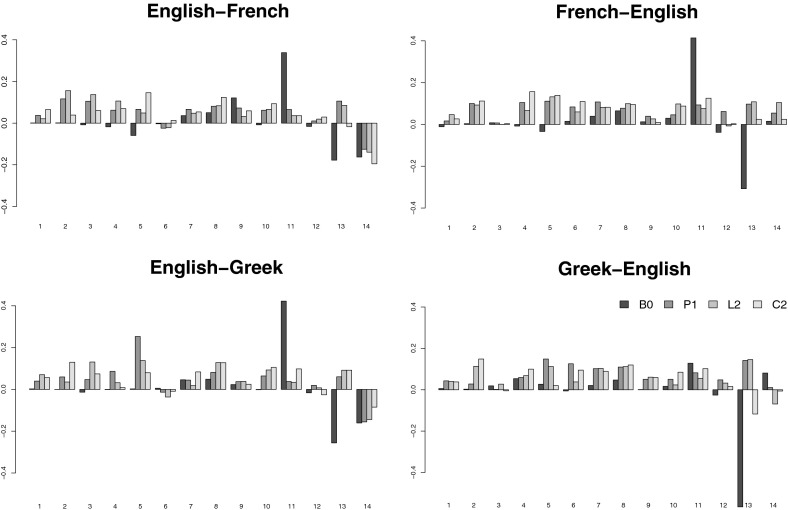
Visualisation of model weights of the systems presented in Table 14 (*env* domain only) based on general-domain data for training and tuning (*B0*), domain-specific parallel data for tuning (*P1*), additional monolingual data for language models (*L2*), and additional parallel data for the translation model (*C2*)

In Fig. 6, we visualise the weight vectors of the four systems presented in this section for the *env* domain (the trends on the *lab* domain are the same). Compared to the baseline (*B0*), the in-domain tuned systems (*P1*) do not trust the translation model that much and prefer hypotheses consisting of more phrases which are shorter and more reordered. The weight vectors of systems *L2* do not change much. A consistent increase, however, is observed for both the language model weight ($h_{8}$) and phrase penalty ($h_{13}$). This is natural, as the language models match the test domain better and the systems are better able to construct improved hypotheses consisting of even shorter phrases. The parameters of the fully adapted systems (*C2*) changed only slightly. A consistent change is observed for the phrase penalty ($h_{13}$); in most cases it dropped, which is reflected in an increase in average phrase length in the test translations compared to systems *L2* (see Table 16).

## Conclusions

In the first main part of the paper (Sect. 4), we focused on a detailed exposition of the pipeline for acquisition of monolingual and parallel data from the World Wide Web. In the second part (Sects. 5–6), we added a thorough investigation of the impact of resources that can be generated using the pipeline, focusing in particular on major established/tried-and-tested approaches to domain adaptation of MT. We discussed the effect of tuning and adaptation on SMT system weights, analysed the learning curves of parameter tuning, OOV rates, perplexity of the test data, and phrase length in translations produced during various stages of adaptation.

The pipeline for the acquisition of domain-specific monolingual and parallel texts from the web is based on existing open-source tools for web crawling, text normalisation and cleaning, language identification, duplicate removal, and parallel sentence extraction. It is implemented as easy-to-use web services ready to be employed in industrial scenarios. It requires only limited human intervention for constructing the domain definition and the list of seed URLs, which can easily be tweaked and tuned to acquire texts with high accuracy of 94 %. This pipeline was applied to acquire domain-specific resources for adaptation of a general-domain SMT system. We crawled monolingual and parallel data for two language pairs (English–French, English–Greek) and two domains (environment, labour legislation), which allowed us to perform a large-scale evaluation using a total of eight test scenarios. The acquired data sets are available from ELRA.

Our domain-adaptation experiments focused on the following three components of a PB-SMT model: parameters of the log-linear combination and their optimisation, language model, and translation model. First, we confirmed the observation from previous research that systems trained and tuned on general domain perform poorly on specific domains. This finding is not surprising, but the amount of loss and the fact that it is observed consistently was rather unexpected. The average absolute decrease in BLEU in all the domain-specific evaluation scenarios was 21.82 points (37.86 % relative).

We confirm the results of previous research on tuning-only adaptation. Tuning the general domain-trained systems on specific target domain data recovers a significant amount of the loss. Several hundreds of sentence pairs used as development data improved the BLEU score of the baseline tuned on general-domain data by 6.64 points absolute (24.82 % relative) on average. A detailed analysis of the model parameters and phrase length distribution in translations of the test data found that a system trained and tuned on general domain data strongly prefers long and few phrases in the output translations, and thus underperforms on specific domains where such phrases do not occur so frequently. In contrast, the same systems tuned on domain-specific data produce output translations from shorter phrases, allow specific reordering and perform significantly and consistently better on specific domains.

Importantly, our findings show that the development data does not have to be manually cleaned and corrected, as parameter tuning on the development set (here, using MERT) is quite tolerant to imperfect translations and eventual noise in the development sets. Cross-domain tuning on a different set also offers a good solution when no in-domain development data is available, especially when the domains differ in a similar way. This step has the effect of tweaking the original general-domain system towards shorter phrases and it does not matter much which different development sets are used.

The experiments with language model adaptation confirmed previous results. Linear interpolation of the general-domain and domain-specific models increased translation quality by a further 4.88 BLEU points absolute (14.95 % relative) compared to the general-domain systems tuned on in-domain development sets on average and significantly outperformed other techniques (concatenation of training data and log-linear combination of the two models). Adaptation of translation models (using 7,000–20,000 acquired sentence pairs) increased BLEU scores by 4.56 points absolute (13.87 % relative) compared to the general-domain systems tuned on in-domain development sets. In this case, linear interpolation and log-linear combination produced similar results. In the combined approach, we observed that the effect of using in-domain monolingual and parallel data is largely independent and does not cancel out when these two types of resources are used at the same time. The final BLEU scores increased by 3.78 points absolute (9.66 % relative) with respect to the language-model-adapted systems, by 8.66 points absolute (26.43 % relative) with respect to the in-domain tuned systems, and by 15.30 points absolute (58.37 % relative) with respect to the general-domain baseline, all on average.

The pipeline for domain-focused web crawling described in this work proved to be very successful in acquisition of domain specific data—both monolingual and parallel. The experiments then showed a high impact of the acquired resources on domain adaptation of MT. We mainly concentrated on parameter tuning and analysis of its effects. Although we did not especially focuse on adaptation of language models and translation models, the acquired data also significantly improved these components and translation quality in general.

## Appendix

### Domain definition: Environment

The environment domain refers to the interaction of humanity and the rest of the biophysical or natural environment. Relevant texts address the impacts of human activity on the natural environment, such as terrestrial, marine and atmospheric pollution, waste of natural resources (forests, mineral deposits, animal species) and climate change. Relevant texts also include laws, regulations and measures aiming to reduce the impacts of human activity on the natural environment and preserve ecosystems and biodiversity, which mainly refer to pollution control and remediation, legislation well as to resource conservation and management. Texts on natural disasters and their effects on social life are also relevant.

### Domain definition: Labour legislation

The labour legislation domain consists of laws, rules, and regulations, which address the legal rights and obligations of workers and employers. Relevant texts refer to issues such as the determination of wages, working time, leave, working conditions, health and safety, as well as social security, retirement and compensation. It also refers to issues such as rights, obligations and actions of trade unions, as well as legal provisions concerning child labour, equality between men and women, work of immigrants and disabled persons. Relevant texts also discuss measures aiming to increase employment and worker mobility, to combat unemployment, poverty and social exclusion, to promote equal opportunities, to avoid discrimination of any kind and to improve social protection systems.

**Table 17:** Complete results of all English–French systems

Domain	System	BLEU	$\varDelta$	1-PER	$\varDelta$	1-TER	$\varDelta$
*env*	*B0*	29.61	0.00	60.46	0.00	42.47	0.00
	*P1*	37.51	7.90	66.28	5.82	51.47	9.00
	*P2*	37.25	7.64	65.96	5.50	51.72	9.25
	*P3*	37.47	7.86	66.12	5.66	51.95	9.48
	*P4*	36.24	6.63	65.32	4.86	50.58	8.11
	*L1*	41.28	11.67	67.91	7.45	54.13	11.66
	*L2*	41.78	12.17	68.23	7.77	54.34	11.87
	*L3*	41.25	11.64	67.99	7.53	54.04	11.57
	*T1*	39.61	10.00	67.40	6.94	53.21	10.74
	*T2*	39.85	10.24	67.67	7.21	53.38	10.91
	*T3*	39.76	10.15	67.58	7.12	53.34	10.87
	*C1*	43.70	14.09	69.14	8.68	55.96	13.49
	*C2*	**43.85**	**14.24**	**69.52**	**9.06**	**56.12**	**13.65**
	*C3*	43.75	14.14	69.49	9.03	55.78	13.31
	*C0*	39.54	9.93	66.72	6.26	52.15	9.68
*lab*	*B0*	23.94	0.00	57.15	0.00	36.40	0.00
	*P1*	32.15	8.21	62.59	5.44	46.87	10.47
	*P2*	31.88	7.94	62.50	5.35	46.03	9.63
	*P3*	31.82	7.88	62.47	5.32	46.03	9.63
	*P4*	30.60	6.66	61.54	4.39	45.14	8.74
	*L1*	36.15	12.21	64.73	7.58	48.83	12.43
	*L2*	38.54	14.60	66.01	8.86	50.70	14.30
	*L3*	35.54	11.60	64.63	7.48	48.40	12.00
	*T1*	41.33	17.39	67.77	10.62	53.07	16.67
	*T2*	42.08	18.14	68.82	11.67	53.71	17.31
	*T3*	42.70	18.76	69.12	11.97	54.06	17.66
	*C1*	47.45	23.51	71.45	14.30	57.97	21.57
	*C2*	**48.31**	**24.37**	**71.94**	**14.79**	**58.89**	**22.49**
	*C3*	47.96	24.02	71.64	14.49	58.57	22.17
	*C0*	43.05	19.11	69.14	11.99	54.63	18.23

$\varDelta$ refers to absolute improvement over the baseline (*B0*)

**Table 18:** Complete results of all French–English systems

Domain	System	BLEU	$\varDelta$	1-PER	$\varDelta$	1-TER	$\varDelta$
*env*	*B0*	31.79	0.00	63.13	0.00	47.79	0.00
	*P1*	39.05	7.26	70.60	7.47	55.64	7.85
	*P2*	38.93	7.14	70.55	7.42	55.55	7.76
	*P3*	38.79	7.00	69.40	6.27	55.21	7.42
	*P4*	34.05	2.26	59.36	−3.77	48.02	0.23
	*L1*	40.58	8.79	71.13	8.00	56.65	8.86
	*L2*	42.63	10.84	71.85	8.72	57.83	10.04
	*L3*	39.93	8.14	70.83	7.70	56.16	8.37
	*T1*	41.08	9.29	71.04	7.91	56.86	9.07
	*T2*	41.92	10.13	71.63	8.50	57.55	9.76
	*T3*	41.65	9.86	71.69	8.56	57.24	9.45
	*C1*	43.93	12.14	72.71	9.58	58.95	11.16
	*C2*	**44.22**	**12.43**	72.69	9.56	**59.00**	**11.21**
	*C3*	44.12	12.33	**72.79**	**9.66**	58.98	11.19
	*C0*	37.86	6.07	68.71	5.58	53.58	5.79
*lab*	*B0*	26.96	0.00	59.94	0.00	43.04	0.00
	*P1*	33.48	6.52	66.60	6.66	50.41	7.37
	*P2*	33.34	6.38	66.55	6.61	50.33	7.29
	*P3*	33.07	6.11	66.58	6.64	50.90	7.86
	*P4*	29.69	2.73	56.85	−3.09	43.88	0.84
	*L1*	38.05	11.09	68.74	8.80	53.54	10.50
	*L2*	41.11	14.15	70.19	10.25	55.42	12.38
	*L3*	33.95	6.99	60.46	0.52	47.78	4.74
	*T1*	43.54	16.58	72.08	12.14	57.50	14.46
	*T2*	45.06	18.10	73.28	13.34	59.03	15.99
	*T3*	45.12	18.16	73.30	13.36	59.03	15.99
	*C1*	50.07	23.11	75.34	15.40	62.66	19.62
	*C2*	**50.56**	**23.60**	**75.71**	**15.77**	**63.13**	**20.09**
	*C3*	50.34	23.38	**75.71**	**15.77**	63.10	20.06
	*C0*	43.74	16.78	72.07	12.13	57.75	14.71

$\varDelta$ refers to absolute improvement over the baseline (*B0*)

**Table 19:** Complete results of all English–Greek systems

Domain	System	BLEU	$\varDelta$	1-PER	$\varDelta$	1-TER	$\varDelta$
*env*	*B0*	21.20	0.00	52.75	0.00	36.76	0.00
	*P1*	27.56	6.36	57.65	4.90	43.48	6.72
	*P2*	27.29	6.09	57.25	4.50	44.00	7.24
	*P3*	27.26	6.06	57.04	4.29	44.44	7.68
	*P4*	27.16	5.96	57.03	4.28	43.94	7.18
	*L1*	33.59	12.39	61.07	8.32	47.48	10.72
	*L2*	34.89	13.69	61.52	8.77	49.82	13.06
	*L3*	33.65	12.45	60.98	8.23	47.74	10.98
	*T1*	30.73	9.53	58.68	5.93	44.91	8.15
	*T2*	30.74	9.54	58.99	6.24	45.29	8.53
	*T3*	31.89	10.69	59.71	6.96	46.18	9.42
	*C1*	**38.41**	**17.21**	**63.99**	**11.24**	51.09	14.33
	*C2*	37.90	16.70	63.27	10.52	**51.61**	**14.85**
	*C3*	38.22	17.02	63.89	11.14	51.28	14.52
	*C0*	29.84	8.64	57.15	4.40	42.89	6.13
*lab*	*B0*	24.04	0.00	53.69	0.00	38.79	0.00
	*P1*	30.07	6.03	59.66	5.97	46.17	7.38
	*P2*	30.23	6.19	59.67	5.98	46.15	7.36
	*P3*	29.68	5.64	57.71	4.02	44.95	6.16
	*P4*	29.76	5.72	58.73	5.04	45.59	6.80
	*L1*	35.09	11.05	62.35	8.66	49.55	10.76
	*L2*	34.15	10.11	61.90	8.21	48.74	9.95
	*L3*	34.33	10.29	61.95	8.26	48.78	9.99
	*T1*	30.48	6.44	60.11	6.42	46.79	8.00
	*T2*	30.51	6.47	60.19	6.50	46.63	7.84
	*T3*	30.51	6.47	59.99	6.30	46.43	7.64
	*C1*	34.29	10.25	62.03	8.34	49.08	10.29
	*C2*	**34.76**	**10.72**	**62.40**	**8.71**	**49.74**	**10.95**
	*C3*	34.48	10.44	61.77	8.08	48.58	9.79
	*C0*	26.19	2.15	55.05	1.36	40.57	1.78

$\varDelta$ refers to absolute improvement over the baseline (*B0*)

**Table 20:** Complete results of all Greek–English systems

Domain	System	BLEU	$\varDelta$	1-PER	$\varDelta$	1-TER	$\varDelta$
*env*	*B0*	29.31	0.00	62.98	0.00	49.00	0.00
	*P1*	34.31	5.00	67.62	4.64	52.70	3.70
	*P2*	34.32	5.01	67.40	4.42	52.49	3.49
	*P3*	33.98	4.67	66.59	3.61	52.06	3.06
	*P4*	31.45	2.14	57.50	-5.48	46.29	−2.71
	*L1*	37.03	7.72	68.67	5.69	54.31	5.31
	*L2*	37.57	8.26	68.93	5.95	54.38	5.38
	*L3*	36.55	7.24	68.62	5.64	53.95	4.95
	*T1*	38.35	9.04	69.00	6.02	55.15	6.15
	*T2*	38.12	8.81	69.31	6.33	54.92	5.92
	*T3*	38.66	9.35	69.49	6.51	55.26	6.26
	*C1*	**40.85**	**11.54**	**70.59**	**7.61**	56.82	7.82
	*C2*	40.64	11.33	70.40	7.42	56.85	7.85
	*C3*	40.81	11.50	70.47	7.49	**56.97**	**7.97**
	*C0*	30.71	1.40	62.76	−0.22	47.26	−1.74
*lab*	*B0*	31.73	0.00	64.48	0.00	51.39	0.00
	*P1*	37.57	5.84	69.53	5.05	54.93	3.54
	*P2*	37.68	5.95	69.39	4.91	55.22	3.83
	*P3*	37.58	5.85	69.21	4.73	55.55	4.16
	*P4*	34.95	3.22	61.40	−3.08	50.20	−1.19
	*L1*	40.15	8.42	70.44	5.96	56.82	5.43
	*L2*	40.09	8.36	70.50	6.02	56.45	5.06
	*L3*	40.01	8.28	70.53	6.05	56.48	5.09
	*T1*	38.07	6.34	69.78	5.30	55.25	3.86
	*T2*	38.20	6.47	69.67	5.19	55.26	3.87
	*T3*	37.90	6.17	69.52	5.04	55.05	3.66
	*C1*	40.69	8.96	70.67	6.19	56.77	5.38
	*C2*	**40.75**	**9.02**	**70.89**	**6.41**	**57.04**	**5.65**
	*C3*	40.62	8.89	70.76	6.28	56.91	5.52
	*C0*	29.54	−2.19	61.76	−2.72	46.52	−4.87

$\varDelta$ refers to absolute improvement over the baseline (*B0*)

## Footnotes

1
http://www.panacea-lr.eu/.

2
http://combine.it.lth.se/.

3
http://crawler.archive.org/.

4
http://nutch.apache.org/.

5
http://openbixo.org/.

6
http://www.httrack.com/.

7
http://eurovoc.europa.eu/.

8
http://hadoop.apache.org/.

9
http://registry.elda.org/services/160.

10
http://dmoz.org/.

11
http://dir.yahoo.com/.

12
http://tika.apache.org/.

13
http://catalog.elra.info/.

14
http://registry.elda.org/services/127.

15
http://www.statmt.org/europarl/.

16
http://www.statmt.org/wpt05/.

17
http://www.statmt.org/wmt11/.

18 Note that the minimum number of development sentences is not strictly given, although we address this issue in Sect. 5.5. The only requirement is that the optimisation procedure (MERT in our case) must converge, which might not happen if the set is too small or somehow unbalanced.
